# Application of analysis of variance to determine important features of signals for diagnostic classifiers of displacement pumps

**DOI:** 10.1038/s41598-024-56498-0

**Published:** 2024-03-13

**Authors:** Jarosław Konieczny, Waldemar Łatas, Jerzy Stojek

**Affiliations:** 1 Department of Process Control, Faculty of Mechanical Engineering and Robotics, AGH University of Krakow, al. A. Mickiewicza 30, 30‐059 Krakow, Poland; 2 Department of Applied Mechanics and Biomechanics, Faculty of Mechanical Engineering, Cracow University of Technology, al. Jana Pawla II 37, 31-864 Krakow, Poland

**Keywords:** Learning systems, Machine learning, Diagnostics, Signal analysis, Multi-piston pump, Vibration, Analysis of variance, Mechanical engineering, Statistics, Information technology

## Abstract

This paper presents the use of one-way analysis of variance ANOVA as an effective tool for ranking the features calculated from diagnostic signals and evaluates their impact on the accuracy of the machine learning system's classification of displacement pump wear.The first part includes a review of contemporary diagnostic systems and a description of typical damage of multi-piston displacement pumps and Its causes. The work also contains description of a diagnostic experiment which was conducted in order to obtain the matrix of vibration signals and the matrix of pressures measured at selected locations on the pump housing and at the pump pressure line. The measured signals were subjected to time–frequency analysis. The features of signals calculated in the time and frequency domains were ranked using the ANOVA. The next step involved the use the available classifiers in pump wear evaluation, conducting tests and assessing their effectiveness in terms of the ranking of features and the origin of diagnostic signals.

## Introduction

Hydraulic drive and control have been used in many fields of technology. This was determined by their many favourable properties, such as: high output power density compared to the dimensions and weight of such systems, ease of automation, negligible moments of inertia of pumps and hydraulic motors, and, in most cases, their self-lubrication. The hydrostatic drive is commonly used in mobile machines, i.e., construction and agricultural machines. Power hydraulics have found application in heavy industry in press systems, rolling mills, pressure die casting machines and mining. Maintaining the operability of hydraulic systems requires keeping a given class of cleanliness of the working fluid and monitoring the physical parameters of their main components (including positive displacement pumps). This is the main task of maintenance engineering. The development of damage in a hydraulic element is usually accompanied by an increase in the temperature of the working fluid in the control signal lines and an increase in pressure pulsation and noise level. Monitoring of machines containing hydraulic systems, which is well-planned and carried out, allows for earlier determination of the working state of system components, thus facilitating the possibility of carrying out planned service works and preventing failures and resulting downtimes. Since hydrostatic drives started to be used in machines and devices, attempts have been made to predict and assess the efficiency of their components with the use of various methods^1^. Examples of their implementation in the diagnosis of hydraulic components are presented below, with particular emphasis on techniques using the so-called intelligent fault identification.

The first, basic and well described in the literature tool used in machine diagnostics is the use of time and time–frequency analysis of signals measured in characteristic places of the tested system (or checked element). Such a solution was used at least in works^2–4^ where the development of abrasive wear of the cam disk in a multi-piston positive displacement pump was monitored. Another example may be the use of the Wavelet transform in the diagnosis of the external tightness of the hydraulic cylinder, which is described in the article^5^.

Another method of diagnosing wear of hydraulic elements (or systems) is related to the construction of a model of the diagnosed element, in which the Kalman filter^6^ or the extended Kalman filter^7,8^ is often used as a state observer. Such a solution was described in^8^ as an effective tool for detecting leaks in the impeller of a multi-piston pump and in^7^ for examining changes in the efficiency of a screw pump. The algorithm of the adaptive Kalman filter was used in works^9,10^ to monitor the efficiency of the hydraulic manipulator and to detect the internal leakage of the hydraulic cylinder.

The next (third) group of systems used to diagnose hydraulic systems are systems with elements of the so-called intelligent damage identification based on the use of machine learning and deep learning algorithms^11–13^. In the literature, there are many papers describing the use of such solutions in the diagnostics of hydraulic systems^14–16^. For example, the possibility of using machine learning in the diagnostics of pumps in liquefied gas regasification installations is presented in^17^. In this case, the condition of the pump was tested based on the prediction of its power demand (power consumption). A comparison of the applied diagnostic models was made using a developed error analysis (minimum relative Error, absolute Error and mean square Error). The best results (the best accuracy) were recorded by the Gradient Boosted Trees GBT model. The following article^18^ presents a combination of the empirical wavelet analysis with the Principal Component Analysis PCA and extreme machine learning, which was used in the diagnostics of a hydraulic piston pump. First, the vibration signals measured in characteristic places of the pump body were decomposed into components of different frequencies using the empirical wavelet analysis. For the signals obtained in this way, features were calculated and then reduced in terms of significance using PCA. The input vector reduced to the most important features was fed to the classifier using an extreme machine learning algorithm. The accuracy of predicting the modelled damage by the built learning system was 100%.

The diagnosis of abrasive wear of the piston foot in an axial piston pump using the extreme machine learning algorithm is presented in the article^19^. Three methods of obtaining features constituting input data to the selected classification models were proposed, i.e. Wavelet Packet Transform (WPT), Empirical Mode Decomposition (EMD) and Local, Mean Decomposition (LMD). The obtained feature vectors were then ranked and the most significant ones were then given to the inputs of the classifiers. Finally, three pump condition prediction models were compared, i.e. Extreme Machine Learning ELM, Back Propagation BP and Support Vector Machine SVM.

The use of the *K*-nearest Neighbors classifier for effective classification of the life state of a multi-piston pump was described in^20^. In turn, the application of the SVM algorithm to monitor the condition of a hydraulic car brake is described in^21^.

Article^22^ presents the use of deep machine learning in the diagnostics of an axial piston pump. Initially, time–frequency portraits were obtained using a continuous Wavelet transform of vibration signals as input data for the Convolutional Neural Networks CNN, then the ranges of the adopted hyperparameters of the network were optimized using the Bayesian optimiser. By integrating deep machine learning and the adaptive Bayesian algorithm, a modified model was obtained for diagnosing failures in pumps.

Article^23^ presents the use of a deep Multi-Signal Fusion Adversarial Model Based Transfer Learning MFAN in the diagnostics of an axial displacement pump operating under variable operating conditions, i.e. variable capacity and pressure (as is the case in most drives of construction and agricultural machines). A modular structure of the system was proposed by first carrying out fusion of vibration and acoustic signals with assigning their weights, with a module for generating features and the adopted structure of the neural network. The average accuracy of MFAN in identifying damage reached 98.5%.

The use of deep machine learning to classify faults in axial piston pumps is also presented in articles^24,25^. Applying the deep belief networks architecture in this case, a high accuracy of classification of the four most common axial piston pump faults was achieved. The classification accuracy was above 97%. Device monitoring with a discussion of Deep Belief Network (DBN), Convolutional Neural Networks (CNN) and Recurrent Neural Networks (RNN) is presented in^26^.

Many papers on the diagnostics of displacement pump failures are based on using previously prepared models of pump component damage. Such an approach does not give the complete picture of the damage and is only its approximation. The authors have applied a different approach, which involves obtaining the pump component wear in a natural way based on a long-term pump operation under an assumed load at lowered oil purity class. In addition to obtaining the image of pump component wear in a natural way, this method allows controlling and observing the damage development and accompanying symptoms.

The review of papers on the diagnostics of hydrostatic systems indicates that most authors evaluating the efficiency of such systems use signals measured in stationary operating conditions (after the system reached thermal stability). The operational practice however proves that information included in signals measured in the non-stationary pump operation (at varying viscosity of the operational liquid) gives a fuller picture of the component wear. To evaluate the pump component wear, the authors have used signals measured in a steady state and in dynamic transient states.

In this paper, the authors have presented the application of machine learning^27^ in the classification of wear of a multi-piston displacement pump. Machine learning systems have many advantages in engineering applications, such as the possibility of building a system of a very good classification accuracy with the use of a reasonable amount of data and short learning times of the developed diagnostic models. From the beginning, the assumption was that the learning system would be based on measures (features) obtained from vibration signals measured at characteristic locations on the pump housing, and additional signals measured on the pump pressure port by static and dynamic pressure transducers which would be obtained during a passive diagnostic experiment. The Choice of measures which were calculated from the obtained signals and the subsequent ranking of their significance was carried out using the analysis of variance (ANOVA)^28,29^.

The originality of the approach involves:

- possibility of using ANOVA for an effective ranking of diagnostic features and evaluation of the impact of the origin of diagnostic signals on the accuracy of classification of displacement pump wear by the machine learning system;
- designing and experimenting with a way that allows obtaining the pump component wear in a natural way based on many hours of pump operation;
- using the signals measured in the entire pump operation range (i.e. in the stationary and non-stationary operating conditions) to evaluate the degree of pump wear.

After the introduction, the rest of this article follows. In Section "Tested object", the most common failures of multipiston pumps are presented, as well as an example of time–frequency analysis of the vibration signals produced when the wear of the pump's valve plate is depressed. Section "Experimental part" describes the conduct of a test experiment at a laboratory station. Section "Selection of the classification system features" presents the features that were calculated from the measured diagnostic signals. Then, using ANOVA analysis of variance, the obtained features were subjected to ranking of their significance, which is described in Section "Application of ANOVA in selection of significant features of measured signals". After discussing in Section "Selection of classification algorithm" the classification algorithms used, the results obtained from the classification of the wear condition of the pump tested by the learning system are presented in Section "Pump wear classification". The article is concluded with a summary.

## Tested object

The tested object was a multi-piston axial pump^30,31^ with a swashplate whose detailed structural diagram is presented in^20^.

As presented in^20^ and^32,33^, the most frequent type of wear in displacement pumps is abrasive wear. Excessive load on the rotor unit among others leads to abrasive wear of its elements and increasing radial clearance within piston-cylinder pairs. It results in increasing volumetric loss and reduced general efficiency of the pump.

**Figure 1:**
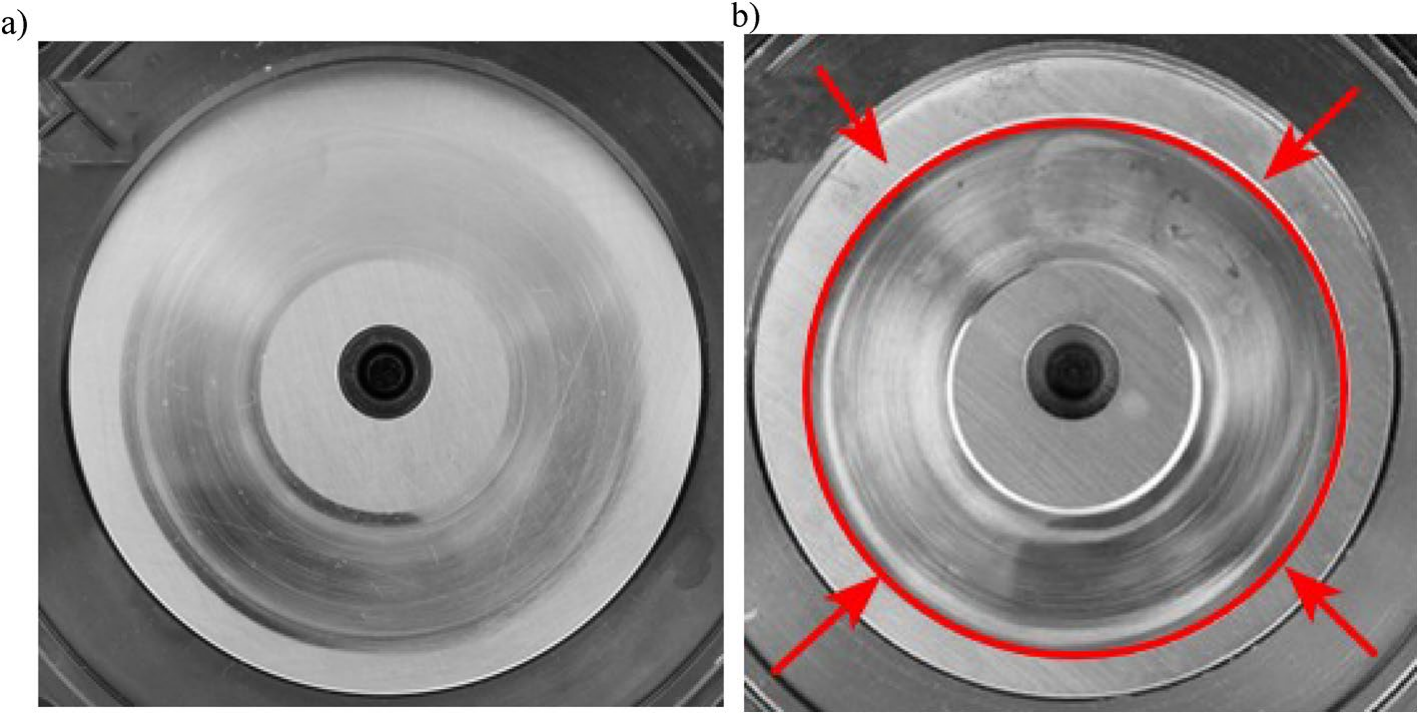
Pump swashplate: (**a**) swashplate in good working order, (**b**) worn swashplate (elliptical notch is visible).

**Figure 2:**
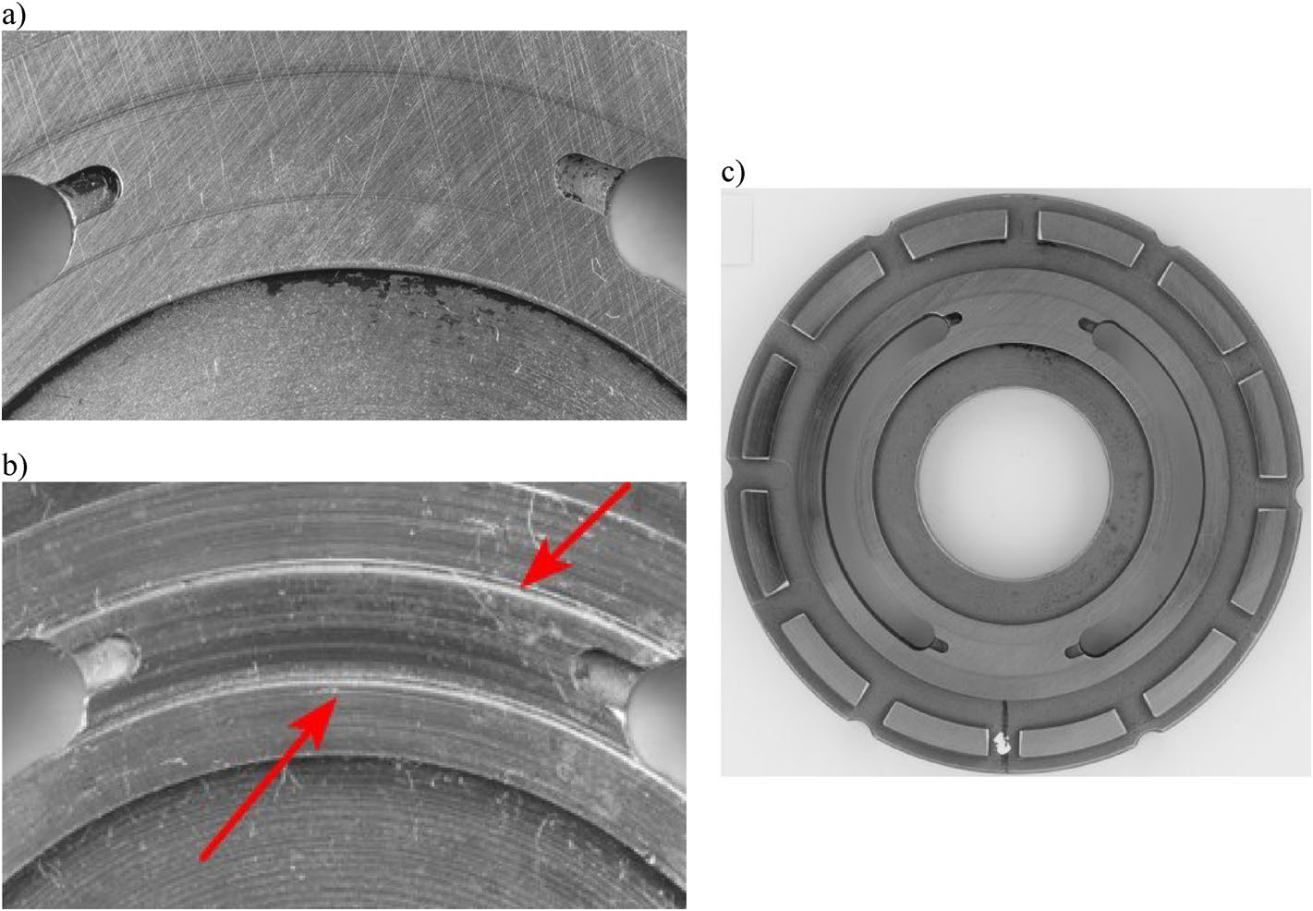
Pump valve plate: (**a**) plate in good working order, (**b**) worn plate (micro-conduits are visible), c) general view.

The wear of the swashplate (Fig. 1) which mates with surfaces of rotor piston shoes, leads to occurrence of elliptical notch (Fig. 1) on its surface and as a consequence to a total wear of this surface. It causes reduction of pump mechanical and hydraulic efficiency. On the other hand, the wear of the valve plate is among other things caused by the decay of the lubrication layer between the disc surface and a surface of the rotor face. It results in occurrence of flow micro-conduits (Fig. 2) on the surface of the plate bridge. Such conduits cause flow of the working medium between suction and pressure zones in the pump, and consequently loss of tightness, reduction of operational pressure and volumetric efficiency of the pump.

The developing wear of the pump components can be traced with the use of conventional diagnostic methods which are based on time and time–frequency analysis of the measured signals. When damage of a pump part is initiated, the measured signal (e.g. vibration signal) starts to include synchronous components, as well as a number of quasi-periodic and chaotic components. We can use traditional techniques described in^3^ to analyze the measured signal and assume that the transition function is linear. When we assume that the transition function is nonlinear, we can use the methods based on deterministic chaos^34^. An example of time–frequency analysis from vibration acceleration signals measured on the pump body (in three directions X, Y, Z) in the development of the swashplate damage which involved gradual deepening of the elliptical notch, is shown in the figures below.

**Figure 3:**
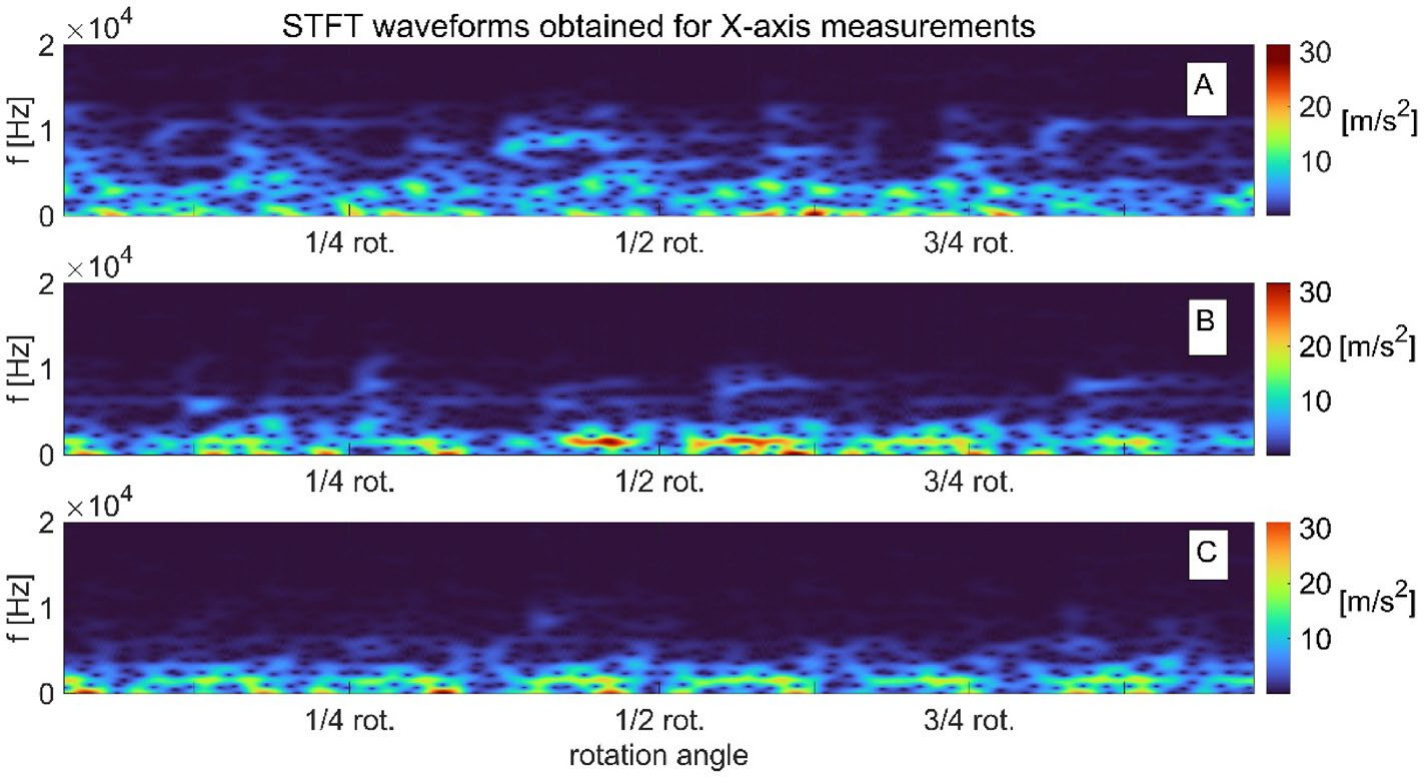
STFT time–frequency representation of vibration signals measured on the pump body in axis X for: (**a**) swashplate without notch, (**b**) swashplate with notch 0.1 mm deep, (**c**) swashplate with notch 0.5 mm deep.

**Figure 4:**
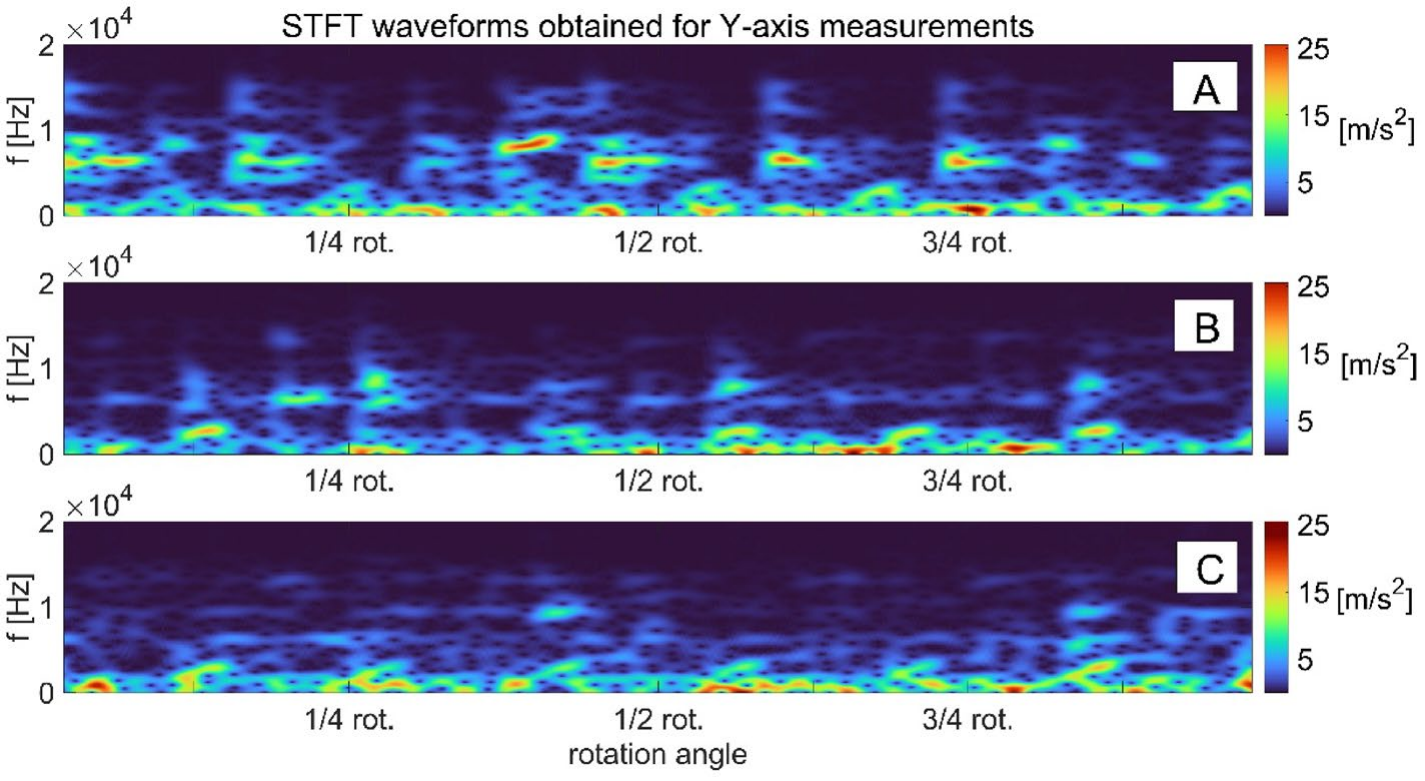
STFT time–frequency representation of vibration signals measured on the pump body in axis Y for: (**a**) swashplate without notch, (**b**) swashplate with notch 0.1 mm deep, (**c**) swashplate with notch 0.5 mm deep.

**Figure 5:**
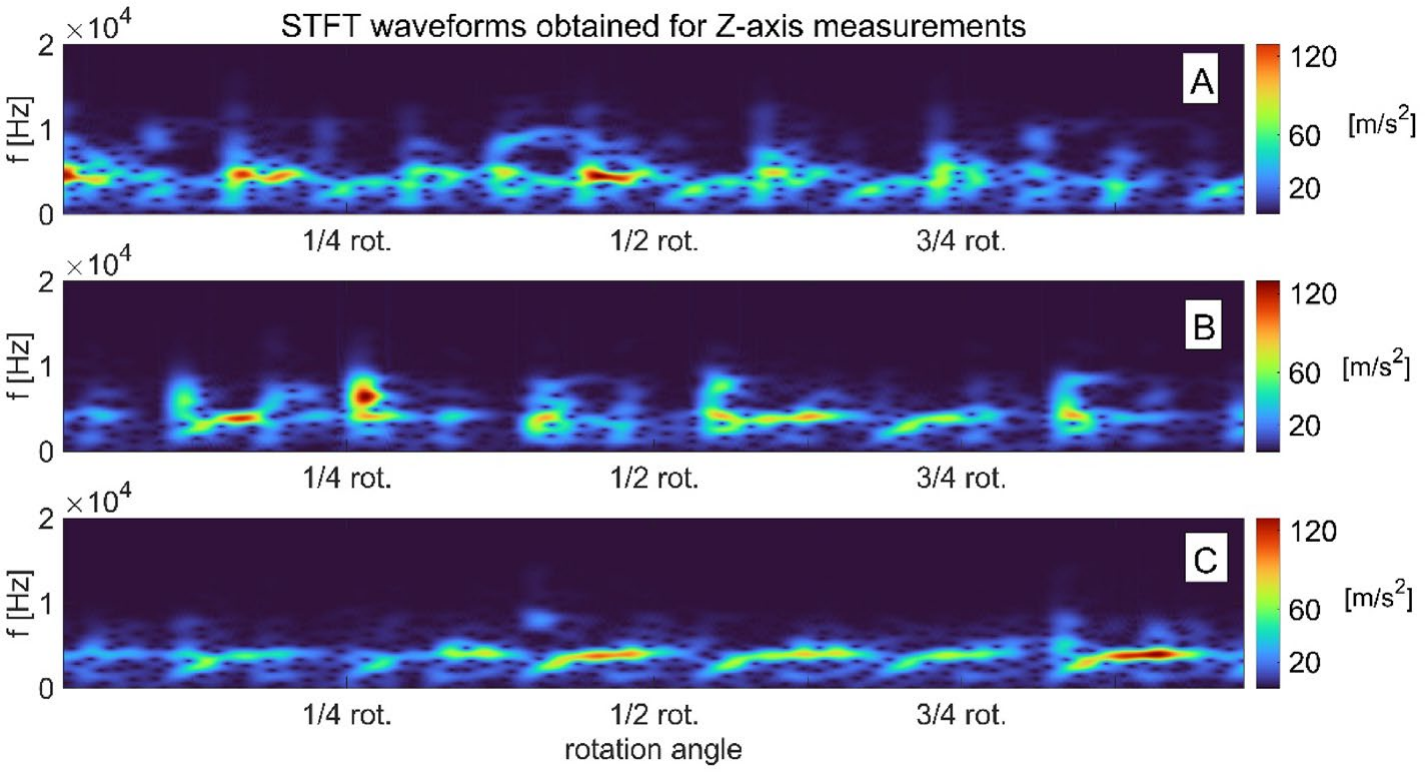
STFT time–frequency representation of vibration signals measured on the pump body in axis Z for: (**a**) swashplate without notch, (**b**) swashplate with notch 0.1 mm deep, (**c**) swashplate with notch 0.5 mm deep.

The analysis of the magnitude squared of the short-time Fourier transform STFT images (called spectrograms) obtained in axes *X* and *Y* (Figs. 3 and 4) indicates that as the swashplate wear increases. The signal spectrum gradually moves towards lower frequencies and its amplitude increases. For the measurements obtained in the X direction, the range of frequency changes of the signal spectra ranges from 4 to 0.02 kHz, and for the signals measured in the Y direction, it is wider, ranging from 10 to 0.02 kHz. In the case of vibration signals from axis *Z* (Fig. 5), there is no clear shift towards lower or higher frequencies, but there is a tendency to reduce the signal’s amplitude as the wear develops. In this direction of measurement (Z direction), the frequency variation range of the signal spectra was 8 to 1 kHz.

## Experimental part

The multi-piston components' wear tests were conducted on a purpose-built laboratory station. One of the main test objectives was to obtain the wear of the pump components in a natural way, hence the multi-hour tests were done under actual operating conditions of the pump. During the test, the diagnostic signals from the installed measuring transducers were measured:

- static pressure;
- dynamic pressure;
- vibration acceleration on the pump body in three axes (X, Y, Z).

**Figure 6:**
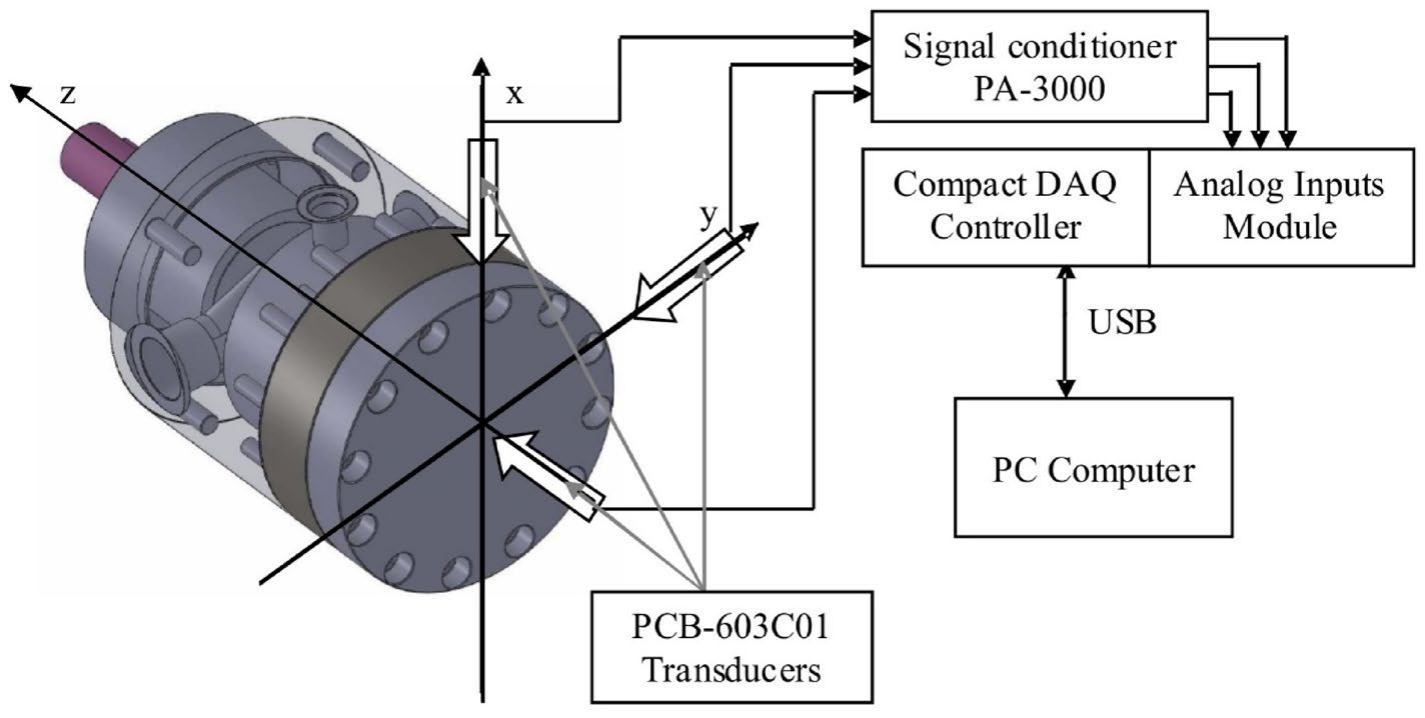
Setup for measuring vibration acceleration on the pump swashplate in axes X, Y and Z of laboratory station.

The acceleration was measured on the pump body in the vicinity of the valve plate and the swashplate. The static and dynamic pressures were measured on the pressure line directly on the outlet port. The sampling frequency of the measured signals was 50 kHz. A simplified diagram of the measuring setup and location of the measuring transducers is presented in Fig. 6.

During the signal measurement, the pump shaft revolution signal was also measured, and each recorded signal was divided by it. In the case of the pump running at nominal speed (n = 1500 rpm), we obtained 25 data series per second of measurement and tables of signals one-revolution long (that is 0.04 s) which corresponded to 2010 measurement points. The 1-s signals were recorded at 15-min intervals between successive measurements. The daily duration of the experiment was 10 h of pump operation under a static load.

The pump operation was monitored by measuring the pressure change at its outlet. By comparing day-by-day pressure values (for individual measurements), it was assumed that in the case when the pressure drop on the pump outlet reaches 10 percent of the load pressure (which was 7 MPa), the pump remains in good working order (classifier: pump operable). When the pressure drop on the outlet further increases (up to 20% of the initial pressure), the pump is classified as close to its end of life (classifier: end of life). When the pressure drop on the pump outlet exceeds 20 percent, the pump is classified as worn (classifier: pump worn). In total, recorded were 441 measured signals (pump body vibration, static and dynamic pressures), of which 294 (evenly 147) were signals for pump in good working order (classifier: pump operable) and pump in a transition state (classifier: end of life). The last 147 signals were from the worn pump (classifier: pump worn). The next stage in preparation of data for the pump condition classification system involved dividing the data into those used for the system learning and the data used for subsequent verification and validation of the system. It was assumed that 30% of the total data would be used to validate and test the obtained classifier, and the remainder (70%) of the real data would be used in the learning process. The prepared data were entered into the Matlab^35^ package, where further analysis occurred. The analysis involved:

- selection and calculation of appropriate signal features;
- ranking of calculated features in terms of information they contain;
- accepting appropriate classifiers;
- evaluation of selected classifiers in the diagnostics of pump condition, in terms of choice of diagnostic signals and the applied feature ranking algorithm.

## Selection of the classification system features

The following significant problem in the construction of the classification system is the selection of signal features on which the system will be based. The features of signals from the time and frequency domains were determined for each obtained matrix of the pump body vibration signals^36^. The dimension of the vibration signal matrix for which the features were calculated was then 3 × 2010 measurement points. The procedure was the same in analysing signals from the static and dynamic pressure transducers installed in the pump pressure port. In this case, the dimension of the signal matrix for which the features were calculated was 2 × 2010 measurement points. In the case of the determination of time features of the measured signals, we defined their variability and the amount of information they contained. We used the statistical measures of location, concentration and variability. The list of features of the measured signals and relationships according to which they were calculated in presented in Table 1.^37^

**Table 1:** The list of features of the measured signals.

No	Expression	Feature	Description
1	$\overline{x }=\frac{1}{n}\sum_{i=1}^{n}{x}_{i}$	Mean	Sum of all data divided by the number of data
2	$\updelta =\sqrt{{\updelta }^{2}}$ where ${\delta }^{2}=\frac{1}{n-1}\sum_{i=1}^{n}{\left({x}_{i}-\overline{x }\right)}^{2}$	Standard deviation	Square root of variance; the variance was estimated using a consistent and unbiased estimator
3	${x}_{RMS}=\sqrt{\frac{1}{n}\sum_{i=1}^{n}{{x}_{i}}^{2}}$	Root mean square	Square root of arithmetic mean of data squared
4	${x}_{kurt}=\frac{\frac{1}{n}\sum_{i=1}^{n}{\left({x}_{i}-\overline{x }\right)}^{4}}{{\delta }^{4}}$	Kurtosis	Measure of the shape of feature distribution
5	${x}_{skw}=\frac{\frac{1}{n}\sum_{i=1}^{n}{\left({x}_{i}-\overline{x }\right)}^{3}}{{\delta }^{3}}$	Skewness	Defines the degree to which a distribution differs from the normal distribution
6	${x}_{sf}=\frac{{x}_{RMS}}{\frac{1}{n}\sum_{i=1}^{n}\left|{x}_{i}\right|}$	Shape factor	Root mean square of the signal divided by the mean value of the signal
7	${x}_{if}=\frac{max\left(\left|{x}_{i}\right|\right)}{\frac{1}{n}\sum_{i=1}^{n}\left|{x}_{i}\right|}$	Impulse Factor	Maximum absolute value of the signal divided by the mean absolute value of the signal
8	${x}_{crest}=\frac{max\left(\left|{x}_{i}\right|\right)}{{x}_{RMS}}$	Crest factor	Maximum absolute value in data divided by the root mean square of the data
9	${x}_{clear}=\frac{max\left(\left|{x}_{i}\right|\right)}{{\left(\frac{1}{n}\sum_{i=1}^{n}\sqrt{\left|{x}_{i}\right|}\right)}^{2}}$	Clearance factor	Maximum absolute value of the signal divided by the square root of the signal amplitude
10	max PSD	Peak amplitude of PSD	Maximum peak of the power spectral density amplitude
11	max Freq	Peak frequency of PSD	Frequency of the maximum peak of the power spectral density amplitude

*x*_i_ – *i-*th measurement data, *n* – total number of data in measurement.

The frequency measures are those which are universally used in the description of the frequency domain signals: maximum power spectral density (PSD) and the frequency at which the PSD reaches its maximum.

## Application of ANOVA in selection of significant features of measured signals

The calculated features of the signals are the carrier of information about the wear of the monitored pump. In general perception, the more information, the better the discriminating power of the method used to classify sets with different features^29,38^. Although, theoretically, the number of calculated signal features (which constitute input data for the classifier) is unlimited, in practice, the aim is to obtain the minimum number of features that well describe the properties of the tested object. This is conducive to getting a compact model with a good fit.

To improve the efficiency of classifiers, it is required to remove correlated and irrelevant features of signals. This leads to a reduction in the dimensions of the feature matrix and allows for a reduction of the needed computing power. Moreover, the reduction of the input data reduces the model training time and prevents its overtraining when creating classifier models.

The available data reduction methods can be divided into those that only find the most important features while removing insignificant ones (Backward elimination, Forward selection and Random forests can be distinguished here) and those that combine features by means of an appropriate transformation reducing their dimension (Principal Component Analysis (PCA), Factor Analysis (FA), Linear Discriminant Analysis (LDA) belong to this group).

In the present article, an easily interpretable and well-documented ANOVA analysis of variance was used to rank the features of the measured signals.The analysis of variance is based on breaking up the total sum of squares (SST) of variance for all observation results into two components^28^:

- sum of squares describing variability within the group (SSE),
- sum of squares describing variability between groups (SSR).

Mathematically, this operation can be presented as:

1 $$\begin{array}{*{20}c} {\mathop \sum \limits_{i = 1}^{k} \mathop \sum \limits_{j = 1}^{{n_{i} }} \left( {x_{ij} - \overline{x}} \right)^{2} = } & {\mathop \sum \limits_{i = 1}^{k} \mathop \sum \limits_{j = 1}^{{n_{i} }} \left( {x_{ij} - \overline{{x_{i} }} } \right)^{2} + } & {\mathop \sum \limits_{i = 1}^{k} \mathop \sum \limits_{j = 1}^{{n_{i} }} \left( {\overline{{x_{i} }} - \overline{x}} \right)^{2} } \\ {{\text{SST}}} & {{\text{SSE}}} & {{\text{SSR}}} \\ \end{array}$$

where *n*_*i*_ – number of data in the *i-*th group, $\overline{{x }_{i}}$ – arithmetic mean in the *i-*th group, $\overline{x }$ – arithmetic mean in the total *n*-element group, *n*- total number of independent observations ${x}_{ij}$ for $j=\mathrm{1,2},\cdots {n}_{i}$

2 $$\left( {n = \mathop \sum \limits_{i = 1}^{k} n_{i} } \right)$$

where *k –* total number of groups.

The data are the basis for verification of the following null hypothesis:

### H_0_

Means calculated in each group are equal:

$${H}_{0}: {m}_{1}={m}_{2}={\cdots m}_{k}$$

vs the alternative hypothesis ${H}_{1}$:

### H_1_

At least two means from the groups differ.

In the analysis of variance, the variance between groups (SSR) is compared with the variance within groups (SSE). If the ratio of variance between groups to variance within groups is significantly high, it is possible to reject the null hypothesis at the accepted level of significance *α* (in our calculations we used *α* = 0.05).

The verification is carried out using the statistical test *F* (Fisher-Snedecor test)^28,29^:

3 $$F = \frac{{ SSR_{k - 1} }}{{SSE_{N - k} }} = \frac{MSR}{{MSE}}$$

where *MSR –* root mean square of deviations between groups:

4 $$MSR = \frac{SSR}{{k - 1}}$$

*MSE* – root mean square of deviations within group:

5 $$MSE = \frac{SSE}{{n - k}}$$

where *k*- number of groups; *n*- total number of independent observations.

Test *F* is used to rank each feature, rejecting the null hypothesis at the accepted level of significance. The low value of *p* – test statistics indicates that the analyzed feature is important for pump wear evaluation.

**Figure 7:**
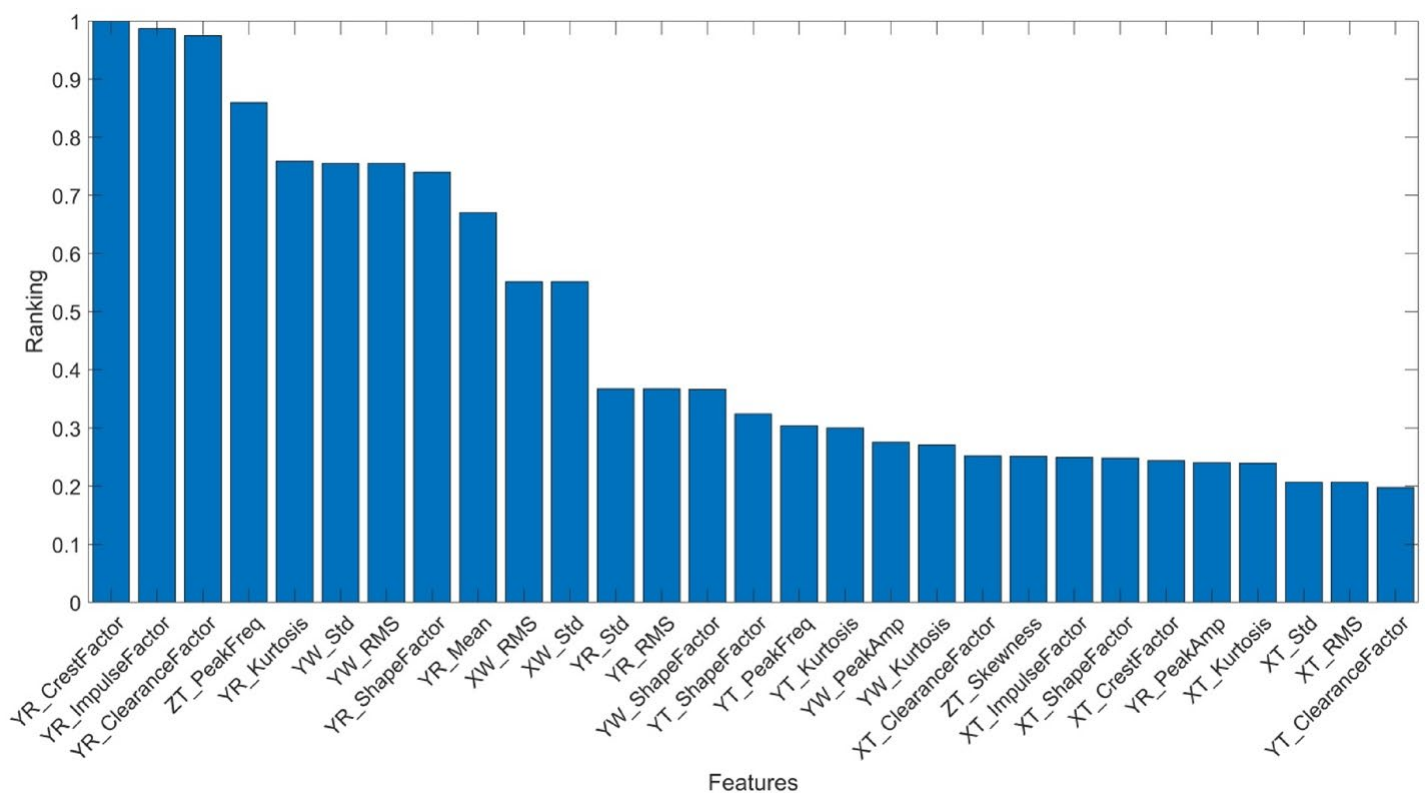
Ranking of feature significance based on ANOVA – vibration signals.

**Figure 8:**
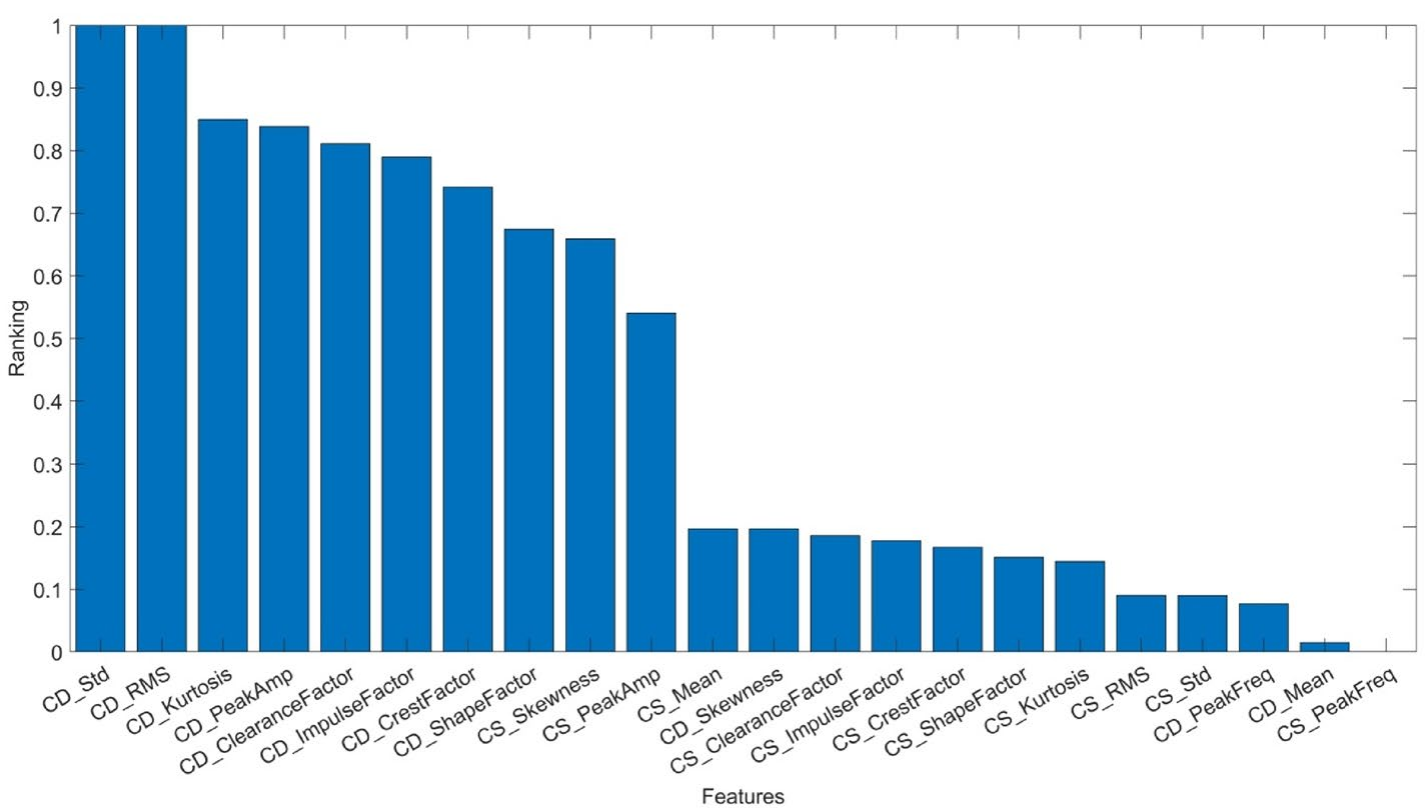
Ranking of feature significance based on ANOVA – pressure signals.

The ranking of significance obtained with ANOVA from the vibration and pressure signals is presented in Fig. 7 (vibration signals) and Fig. 8 (pressure signals).

From among 62 features which were obtained from the pump body vibration measurement, the five most significant ones were selected to evaluate pump wear. The ANOVA ranking of the most significant features is presented in Table 2.

**Table 2:** The ranking of the most significant features obtained from the pump body vibration measurement.

No	Feature	Ranking
1	*YR_CrestFactor*	1
2	*YR_ImpulseFactor*	0.98
3	*YR_ClearanceFactor*	0.97
4	*ZT_PeakFreq*	0.85
5	*YR_Kurtosis*	0.75

where: *YR_CrestFactor* – crest factor of vibration signal measured on the valve plate in axis Y, *YR_ImpulseFactor* – impulse factor of vibration signal measured on the valve plate in axis Y, *YR_ClearanceFactor* – clearance factor calculated from the vibration signal measured on the valve plate in axis Y, *ZT_PeakFreq* – frequency of maximum power spectral density PSD of vibration signal measured on the valve plate in axis Z, *ZT_Kurtosis* – kurtosis of vibration signal measured on the valve plate in axis Y.

From among 22 features obtained from the measurement of static and dynamic pressures on the pump pressure port, the five most significant ones were selected. The features which satisfy the conditions are presented in Table 3.

**Table 3:** The ranking of the most significant features obtained from the static and dynamic pressures measurement.

No	Feature	Ranking
1	*CD_Std*	1
2	*CD_RMS*	1
3	*CD_Kurtosis*	0.85
4	*CD_PeakAmp*	0.83
5	*CD_ ClearanceFactor*	0.81

where: *CD_Std* – standard deviation of dynamic pressure signal, *CD_RMS* – RMS of dynamic pressure signal, *CD_Kurtosis* – kurtosis of dynamic pressure signal, *CD_PeakAmp* – maximum peak of the power spectral density amplitude from dynamic pressure signal, *CD_ClearanceFactor* – clearance factor of dynamic pressure signal.

## Selection of classification algorithm

The learning systems are increasingly used in maintenance engineering for modelling a state of an industrial process or its component (e.g. a machine) exclusively based on available measurement data assigned to the process (class). In terms of learning techniques, the learning systems are divided into supervised and unsupervised^27,39^.

Both supervised and unsupervised machine learning systems offer a large group of learning algorithms and the Choice of the most appropriate one depends on many factors. Firstly, in order to choose the appropriate learning algorithm, we need to specify the task that the model will perform accurately (classification, regression, grouping). The next issue is the type and size of input data which affect the learning speed, load on the computer (controller) memory and accuracy of output data prediction (model answers). The choice of appropriate classification algorithm is not clear-cut and only an experienced operator (a long-standing researcher) can quickly indicate the appropriate algorithm. Usually, the best classification algorithm is selected in multiple trials of individual types and evaluation of the obtained classifiers in terms of speed of operation, accuracy of classification and load on the memory of the computing unit.

Among the algorithms which meet the issue^25^ of the multi-piston pump condition classification we can find:

### Decision trees

In this algorithm, data classification is performed using the decision tree based on the starting point and branches forming a binary decision making system whose end branches are the result of assigning the data to a class.

### Discriminant analysis

It is based on the analysis of the Gaussian distribution of signals from the set of observations (inputs). The classifier estimates the Gaussian distribution parameters from observations and based on that assigns to an appropriate class.

### Support vector machines

Classification of data by finding the best hyperplane which separates the data of one class from the data of the other class. The best hyperplane is the one which separates the data with the largest margin.

### *K* Nearest Neighbours classifiers

It determines the membership of new data from the input set in a specific class based on the location of an assumed number (number *K*) of the nearest (neighbouring) data of the input set data relative to that data. The measure of location is measure of distance of the classified data from the neighbouring data.

### Naive Bayes classifiers

A probabilistic classifier which (naively) assumes mutual independence of input data. Using the Bayes’ theorem, this classifier calculates the probability of data membership in a specific class.

Before starting the verification of the suitability of the aforementioned classifiers in the evaluation of pump wear, it was assumed that each classifier using 5 most significant features of measured signals evaluates the pump condition with the same accuracy as the classifier based on previously determined all features of the measured signals (i.e. 64 features determined from vibration signals or 22 features determined form pressure signals).

In order to confirm this assumption, the following null hypotheses should be assumed and verified:

#### Hypothesis *H*_01_

The full model using all 64 features of the measured vibration signals classified the wear condition of a multi-piston pump with the same accuracy as a simplified model using 5 most significant features.

And

#### Hypothesis *H*_02_

The full model using all 22 features of the measured pressure signals classified the wear condition of a multi-piston pump with the same accuracy as a simplified model using 5 most significant features.

The verification of hypotheses *H*_01_ and *H*_02_ was carried out for models using the *K* Nearest Neighbors algorithm. We used a multiple repetition (5 × 2) *t*-*Student* test with a random division of signals. The measure of the accuracy of pump wear classification by the full model and the simplified model was fit error factor *e* specified by the equation:

6 $$e = \frac{{\mathop \sum \nolimits_{j = 1}^{{n_{test} }} w_{j} I\left( {\widehat{{p_{{{\text{1j}}}} }} \ne y_{j} } \right)}}{{\mathop \sum \nolimits_{j = 1}^{{n_{test} }} w_{j} }}$$

where: *n*_*test*_ – number of observations; *w*_*j*_ – weight of the *j-*th observation; *I*(*x*) – function marker; is *1* for true assumption, otherwise is *0*; $\widehat{{\text{p}}_{\text{1j}}}$ – identified pump condition for the first model in the *j*th observation; $y_{j}$ – actual pump condition in the *j-*th observation.

In the comparison of the pump condition classification by the full and simplified models (for features calculated from vibration signals) the evaluation of hypothesis *H*_01_ was zero (*h* = 0 at probability *p* = 0.57). This indicates that hypothesis *H*_01_ cannot be rejected, and, consequently, it is possible to use the simplified model based on the 5 most significant features of vibration signals.

In the comparison of the pump condition classification by the full and simplified models (for features calculated from pressure signals) the evaluation of hypothesis *H*_02_ was also zero (*h* = 0 at probability *p* = 0.1). Also in this case, the hypothesis *H*_02_ cannot be rejected, and, consequently, it is possible to use the simplified model based on the 5 most significant features of pressure signals.

An example of estimation of the classification errors by models using the features calculated from the pressure signals for the multiple repetitions (5 × 2) *t*-*Student* test is presented in the Table 4 and Table 5.

**Table 4:** Calculated errors e1 of pump wear classification for the full model using 22 features from pressure signals.

5 × 2 *t* Test	1	2
1	0.2581	0.3125
2	0.2581	0.5000
3	0.3871	0.3125
4	0.3871	0.4063
5	0.2581	0.2500

**Table 5:** Calculated errors e2 of pump wear classification for the simplified model using 5 most significant features from pressure signals.

5 × 2 t Test	1	2
1	0.4194	0.3750
2	0.3226	0.3438
3	0.2903	0.4063
4	0.2903	0.3438
5	0.2258	0.3750

## Pump wear classification

The pump wear classification was carried out using models based on 5 most significant features of the measured vibration and pressure signals. The following classification algorithms were used^37,38^:

- Decision trees;
- Discriminant analysis;
- *K* Nearest Neighbours classifiers;
- Naive Bayes classifiers;
- Support vector machines.

**Figure 9:**
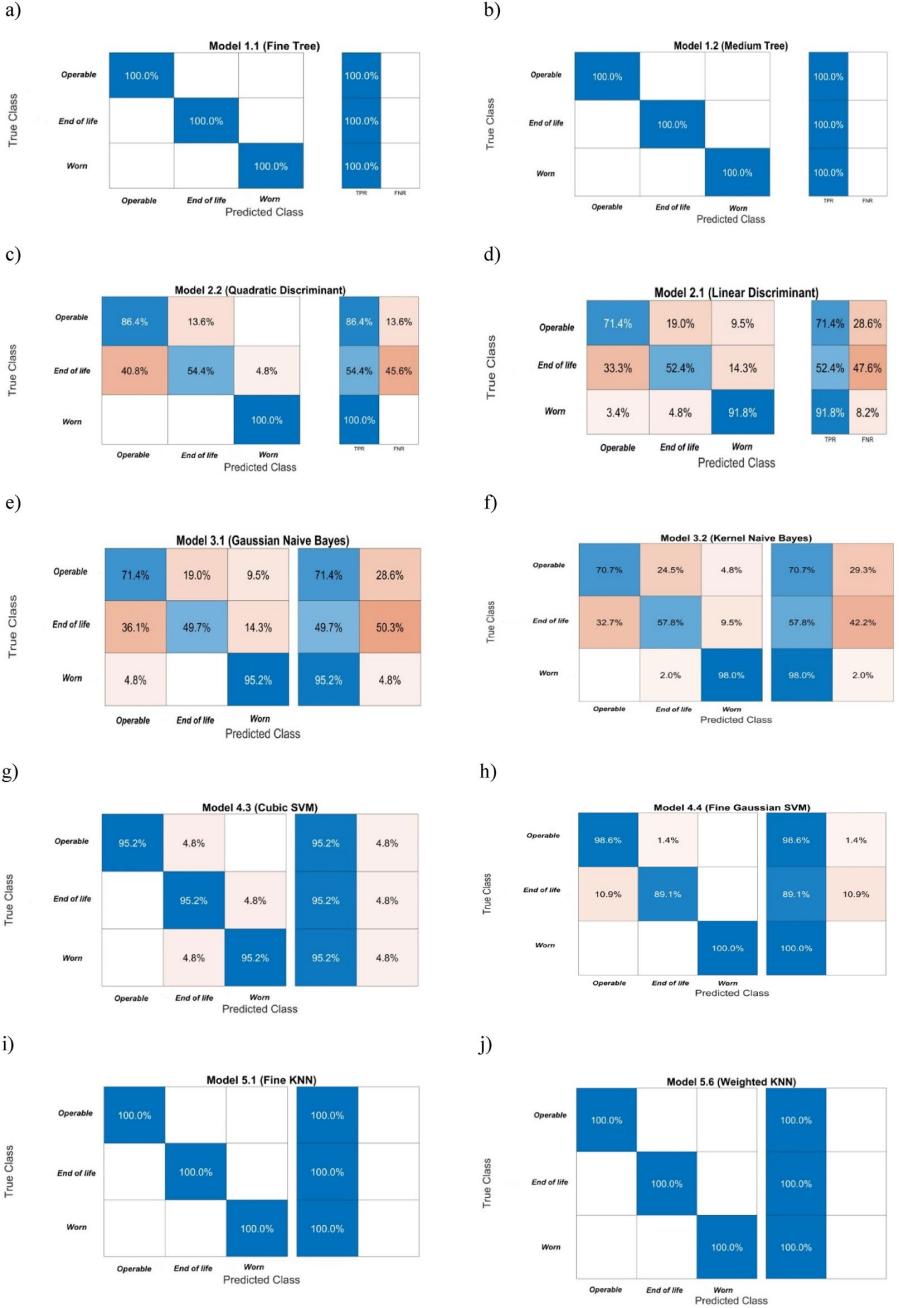
Error matrices for pump condition classification obtained from models using the classifiers: (**a**, **b**) Decision trees, (**c**, **d)** Discriminant analysis, (**e**, **f**) Naive Bayes classifiers, (**g**, **h**) Support vector machines, (**i**, **j)** *K* Nearest Neighbours.

The models' accuracy of the pump condition identification was verified using cross-validation. The input data (calculated values of signal features) were divided into five disjoint sets which were used as test sets, and the remaining used as the learning set. Next the mean Error was calculated. The correctness of pump condition identification by each model was presented graphically as a Confusion Matrix. Figure 9 shows the classification error matrices for the models which as input data use the calculated features from pressure measurements.

Table 6 includes a comparison of properties used to evaluate the pump condition, by the following criteria:

- Classification accuracy;
- Learning time;
- Classification speed;
- Misclassification costs.

**Table 6:** Comparison of properties of classifiers used to evaluate pump wear.

Model	Decision tree		Discriminant analysis		Naive Bayesian		Support vector machines		*K*NN	
	Fine	Medium	Quadratic	Linear	Gaussian	Kernel	Cubic	Fine Gaussian	Fine	Weighted
Prediction speed [obs/s]	5500	5000	11,000	12,000	15,000	5000	4900	3800	6700	7700
Training time [s]	4.6	4.05	1.16	1.36	1.17	2.1	4.49	4.97	1.41	1.47
Total misclassification cost [-]	0	0	87	124	123	108	21	18	0	0
Accuracy [%]	100	100	80.3	71.9	72.1	75.5	92.2	95.9	100	100

**Figure 10:**
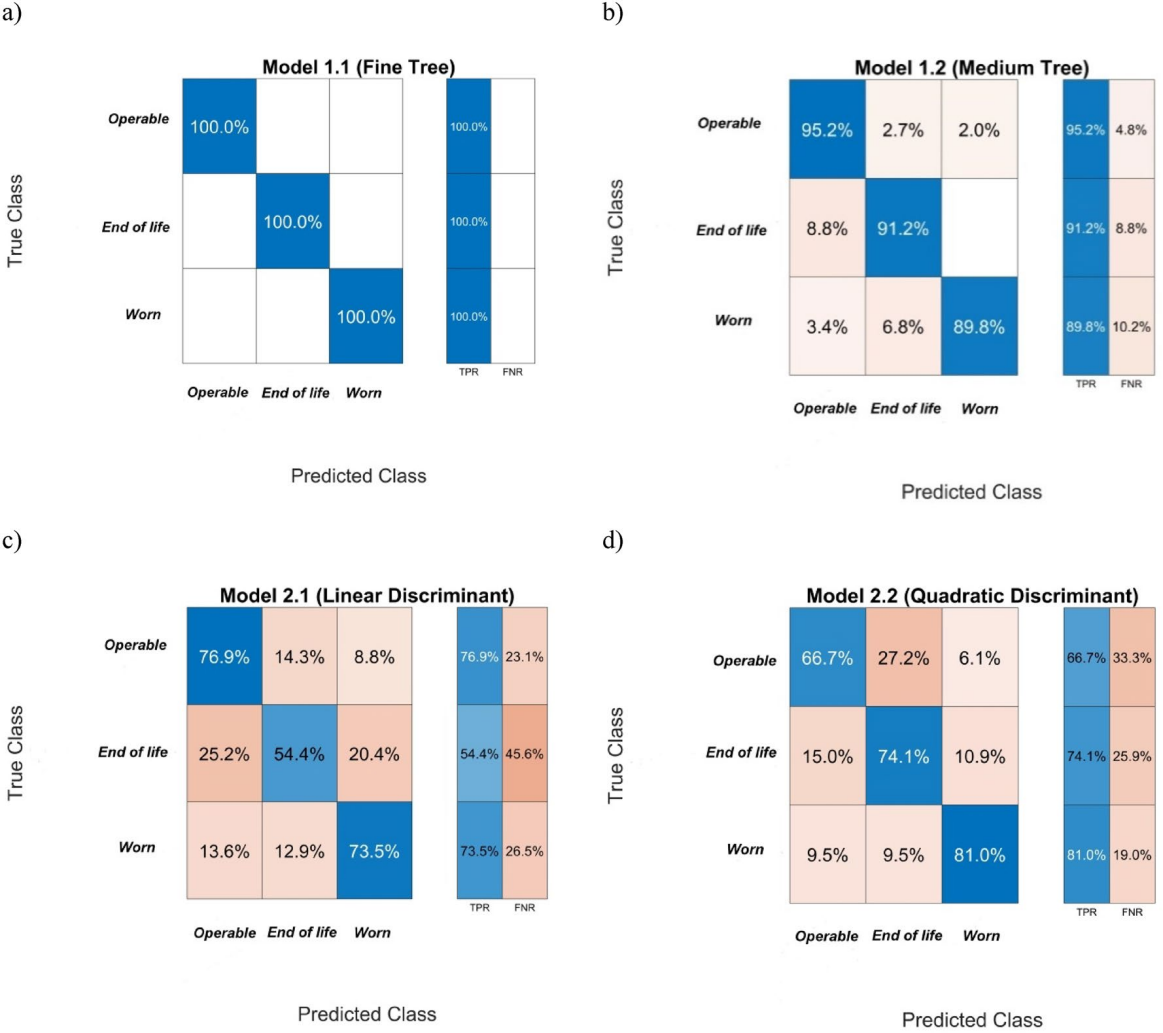
Error matrices for pump condition classification obtained from models using the classifiers: (**a**, **b**) decision trees, (**c**, **d)** discriminant analysis.

**Figure 11:**
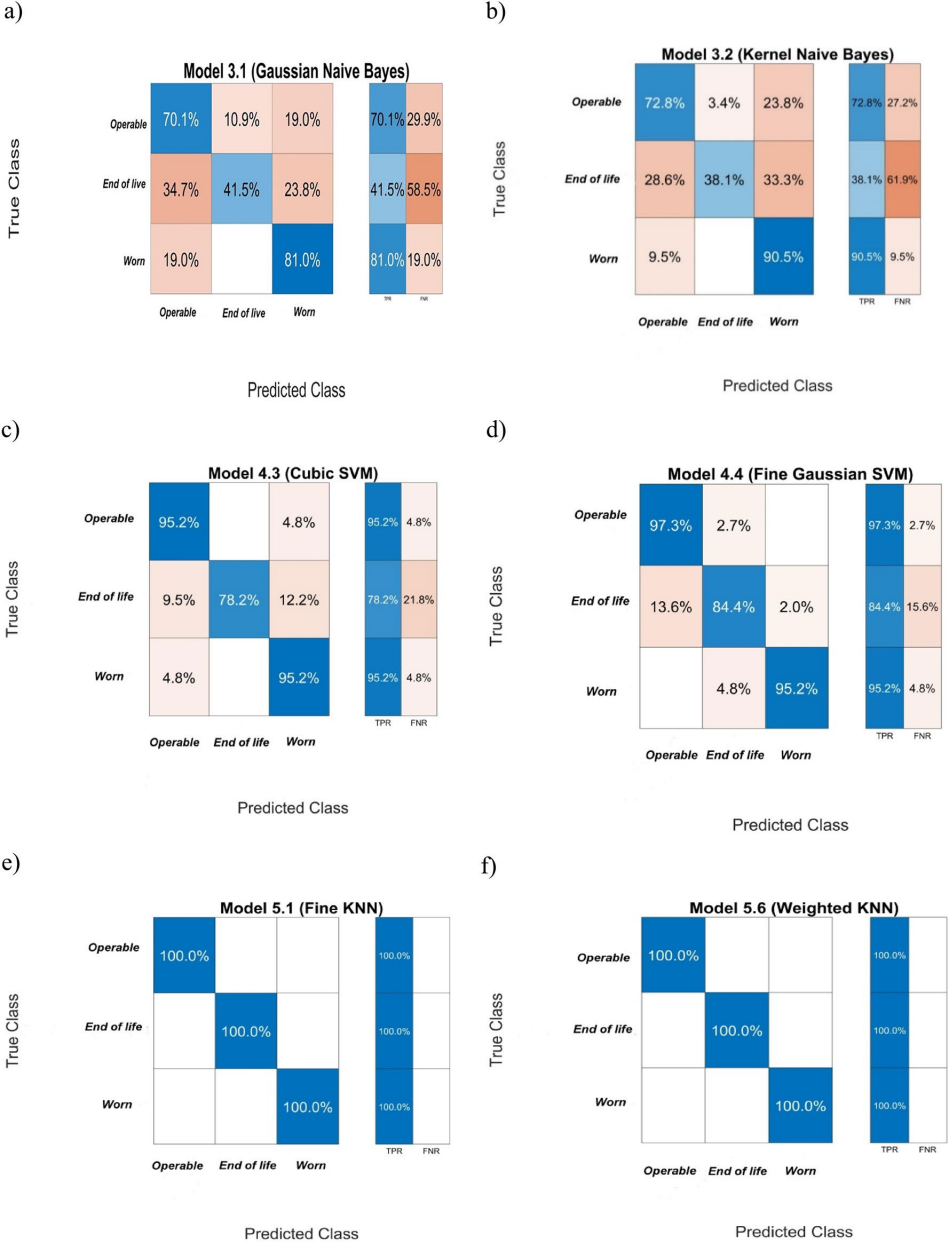
Error matrices for pump condition classification obtained from models using the classifiers:(**a**, **b**) Naive Bayes classifiers, (**c**, **d**) Support vector machines, (**e**, **f**) *K* Nearest Neighbours.

Similarly to the models based on the features obtained from pressure signals, the correctness of the pump condition classification by the models using the most significant features determined from vibration signals was estimated using a Confusion Matrix and is shown in Figs. 10 and 11.

The accuracy of pump condition classification by models and their main features are presented in Table 7.

**Table 7:** The accuracy of pump condition classification by models and their main features.

Model	Decision tree		Discriminant analysis		Naive Bayesian		Support vector machines		*K*NN	
	Fine	Medium	Quadratic	Linear	Gaussian	Kernel	Cubic	Fine Gaussian	Fine	Weighted
Prediction speed [obs/s]	6600	6100	13,000	11,000	12,000	5300	4900	3800	6700	7700
Training time [s]	4.02	3.55	1.14	1.3	1.18	2.06	4.49	4.97	1.41	1.47
Total misclassification cost [-]	0	35	115	140	160	145	21	18	0	0
Accuracy [%]	100	92.1	73.9	63.7	63.7	67.1	92.2	95.9	100	100

The comparison of model properties presented in Tables 6 and 7 indicates that models that use the features from pressure signals had better classification accuracy for each used classification algorithm than the models using the features calculated from vibration signals, while the prediction speed and model learning time were comparable. The 100% accuracy of the pump condition detection was obtained for models using the algorithm *K* Nearest Neighbours *K*NN (regardless of the types of features). The Fine Decision tree models also had high prediction accuracy, but the learning time was on average three times longer than in *K*NN. On the other hand, the models using Support vector machines SVM classified the pump condition with identical accuracy, regardless of input signal type. The models using the Naive Bayes classifiers and Discriminant analysis classifiers had relatively the lowest accuracy of pump condition prediction which was about 70% for the measured pressure input signals and 65% for the measured vibration signals. The classifiers had the largest error in the detection of the pump transition state (*end of life* label), most frequently identifying it as “pump operable”.

A physical implementation of a diagnostic system based on the machine code generated from the selected classification model requires a selection of a model which offers the highest prediction of pump condition and, at the same time, has the least classification error. Based on the comparison of model accuracies presented in Tables 6 and 7 we can say that this condition is satisfied by all *K* Nearest Neighbours models *K*NN and the Weighted *K*NN model (for both pressure and vibration signals). In addition, the Fine Decision tree model has the 100% accuracy for features from the measured pressure and vibration signals. Further analysis of the models mentioned above involved verification of their accuracy for newly measured pressure and pump body vibration signals at three states of operating condition.

Table 8 presents the accuracies with which the flowing models exported to the Matlab space^35^: Fine* K*NN, Weighted* K*NN and Fine Decision tree recognized the pump condition classes based on the previously calculated features.

**Table 8:** The accuracies of the models.

No	Model	Input signal	
		Pressure (%)	Vibration (%)
1	Fine decision tree	94.8	97.7
2	Fine *K*NN	100	100
3	Weighted *K*NN	100	100

**Figure 12:**
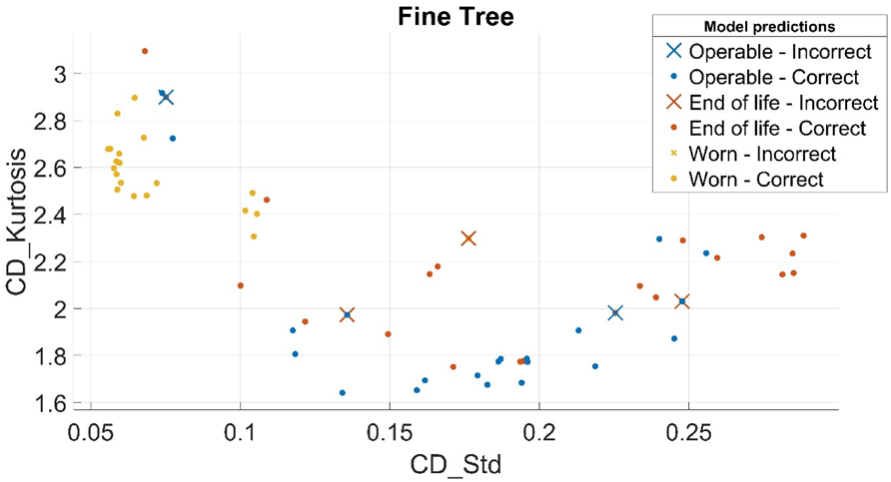
Example of prediction of kurtosis distribution as a function of standard deviation of dynamic pressure signal obtained using verified Fine decision tree model.

An example of the kurtosis distribution as a function of standard deviation of the dynamic pressure signal which was obtained using the verified Fine Decision tree model is shown in Fig. 12.

## Summary

A careful preparation of input data is a key factor in modern systems that use machine learning to classify a monitored machine's wear state. The paper presents a concept of such system using a displacement pump as a diagnosed machine and operational and vibration signals as a source of information. While diagnosing displacement pumps, such data may come from operation signals such as static and dynamic pressures measured on the pump pressure port and additional signals, such as vibration signals recorded in characteristic locations on the pump body. In both cases, the input data sets containing measured signals should be as numerous as possible. In diagnostics of displacement pumps, the input data must include signals in the entire range of the pump operation, i.e. at varying working pressures and with viscosity of the working medium which varies with changing temperature. This results in a better training of the obtained classifier model and better effectiveness of the pump wear classification. The next important issue is the selection of such signal features that best determine the classes of pump condition or damage. It is desirable to determine the minimum number of features which may affect the classifier learning time during the classification process and prevent over-training. ANOVA used in the ranking of the determined features effectively arranged the features coming from signals of both vibration and pressure. The five most significant features were used to evaluate the effectiveness of the accepted pump condition classification algorithms. The obtained results unequivocally indicate that, regardless of the type of the used input features, the best classification accuracy was obtained using the *K* Nearest Neighbours models *K*NN (Fine *K*NN Weighted *K*NN models) and the Decision tree models (Fine Tree models and the Medium Tree model). Support vector machines models (Cubic and Fine Gaussian models) showed slightly worse classification accuracy. In the case of physical implementation in a diagnostic system, in addition to the accuracy with which a given model is able to predict the wear and tear of a pump, the time required to train it must be taken into account. Assuming that the results obtained on a limited number of measured signals can be related to the real operation of the system, the obtained *K* Nearest-Neighbours *K*NN models were trained on average three times faster than the other models that provided equally high prediction accuracy (Decision tree models and Support vector machines models). In turn, the best model recognition speed expressed as the number of recognized observations per second (obs/s) was achieved by the Discriminant analysis models with a lower accuracy of pump state classification.

The authors plan to continue their research on the application of deep machine learning and machine learning systems to the diagnosis of the wear state of positive displacement pumps. At the same time, they would like to extend this research to the detection of the undesirable phenomenon of cavitation, which causes erosive wear of both the suction port and pump components. Based on the results already obtained using machine learning (e.g. the example verification of the Fine Decision tree model shown in Fig. 12), the authors plan to use them to build an advisory system. Such a system would be aimed at hydraulic system operators. The idea is that the system would make use of pressure signals measured at the pump output, from which a decision would be made about the wear state of the pump and the required service.

## Data Availability

The datasets used during the current study available from the corresponding author on reasonable request.
